# Faster indicators of chikungunya incidence using Google searches

**DOI:** 10.1371/journal.pntd.0010441

**Published:** 2022-06-09

**Authors:** Sam Miller, Tobias Preis, Giovanni Mizzi, Leonardo Soares Bastos, Marcelo Ferreira da Costa Gomes, Flávio Codeço Coelho, Claudia Torres Codeço, Helen Susannah Moat

**Affiliations:** 1 Data Science Lab, Behavioural Science, Warwick Business School, University of Warwick, Coventry, United Kingdom; 2 The Alan Turing Institute, London, United Kingdom; 3 Programa de Computação Científica, Fundação Oswaldo Cruz, Rio de Janeiro, Brazil; 4 Escola de Matemática Aplicada, Fundação Getulio Vargas, Rio de Janeiro, Brazil; 5 Institute of Global Health, University of Geneva, Geneva, Switzerland; Center for Disease Control and Prevention, UNITED STATES

## Abstract

Chikungunya, a mosquito-borne disease, is a growing threat in Brazil, where over 640,000 cases have been reported since 2017. However, there are often long delays between diagnoses of chikungunya cases and their entry in the national monitoring system, leaving policymakers without the up-to-date case count statistics they need. In contrast, weekly data on Google searches for chikungunya is available with no delay. Here, we analyse whether Google search data can help improve rapid estimates of chikungunya case counts in Rio de Janeiro, Brazil. We build on a Bayesian approach suitable for data that is subject to long and varied delays, and find that including Google search data reduces both model error and uncertainty. These improvements are largest during epidemics, which are particularly important periods for policymakers. Including Google search data in chikungunya surveillance systems may therefore help policymakers respond to future epidemics more quickly.

## Author summary

To respond quickly to disease outbreaks, policymakers need rapid data on the number of new infections. However, for many diseases, such data is very delayed, due to the administrative work required to record each case in a disease surveillance system. This is a problem for data on chikungunya, a mosquito-borne disease which is a growing threat in Brazil. In Rio de Janeiro, delays in chikungunya cases being recorded average four weeks. These delays are sometimes longer and sometimes shorter. In stark contrast to chikungunya data, data on what people are searching for on Google is available almost immediately. People suffering from chikungunya might search on Google for information about the disease. Here, we investigate whether rapidly available Google data can help generate quick estimates of the number of chikungunya cases in Rio de Janeiro in the previous week. Our model uses a Bayesian methodology to help account for the varying delays in the chikungunya data. We show that including Google search data in the model reduces both the error and uncertainty of the chikungunya case count estimates, in particular during epidemics. Our method could be used to help policymakers to respond more quickly to future chikungunya epidemics.

## Introduction

Chikungunya, a mosquito-borne viral disease or *arbovirosis*, is a growing global public health challenge. In Brazil, there have been over 640,000 reported cases since 2017, with 100,000 to 250,000 cases per year [1]. Nearly a quarter of these cases have been recorded in the state of Rio de Janeiro [1]. In the city of Rio de Janeiro, it is estimated that 18% of the population have already been exposed to the chikungunya virus [2]. By conservative estimates, acute infections lead to chronic health complications in around 25% of cases, such as paralysis and long-term debilitating syndromes [3–6]. Fatality rates may also be higher than previously recognised, due to challenges in determining the cause of death [5]. Chikungunya incidence is highly seasonal, with one epidemic per year during the warmer months when mosquitoes are more active. Epidemics in Ceará, Brazil, have caused major disruptions to their healthcare system [7], and the economic costs from treatment and workplace absence are often catastrophic for the low-income households affected by chikungunya [8].

Disease statistics are prone to delays, as there is often a lag between a patient seeking treatment, being diagnosed, and the case being recorded in disease surveillance databases [9]. In Rio de Janeiro, for chikungunya cases, this delay averages around four weeks, with data arriving gradually and inconsistently. Faster surveillance is therefore strongly desirable, with the goal of allowing public health policymakers to respond more quickly to epidemics [10], facilitating better targeting of mosquito control activities and greater awareness in the general population of the need to take precautions. More up-to-date data would also provide doctors with valuable information when patients present with symptoms that are common across multiple diseases. More broadly, the consequences of failing to respond at sufficient speed to the spread of disease have been emphatically underlined by the Covid-19 crisis [11, 12].

In the absence of faster protocols for recording cases of disease, another option is to augment the available case count data by combining it with other readily available data sources. Online data is a useful source of information for improving the quality and timing of disease surveillance. People experiencing symptoms of a disease may not only consult a medical professional for help, but may also search for information on Google, potentially before seeking professional assistance. In contrast to official case counts, weekly data on Google searches is reliably available with no delay. Previous studies have shown a relationship between internet search data and case counts for diseases such as the flu [13, 14] and dengue [15–17]. Here, we investigate whether Google search data can help generate faster estimates of chikungunya case counts in Brazil.

Estimating the value of statistics before they are officially released is known as “nowcasting”, a term first used to describe real-time estimation of delayed macroeconomic statistics [18]. Nowcasting is strongly related to forecasting: where forecasting seeks to predict the future, nowcasting seeks to “predict the present” [19]. A promising sign that internet search data may help nowcast chikungunya can be found in one study that reports a positive correlation between Google search activity and chikungunya incidence in the Amazon [20]. The question is whether this is a sufficiently strong and consistent relationship to reduce the error and uncertainty of chikungunya nowcasts, in comparison to a model that uses historic chikungunya case data alone.

To investigate this question, we build on a Bayesian approach specifically designed for nowcasting where case data is subject to long and varied delays [17, 21]. Better chikungunya nowcasts could help public health authorities respond more quickly to future epidemics, therefore mitigating their damage [22].

## Materials and methods

### Ethics statement

We use two main data sources in our analyses. The first is anonymised chikungunya case data from Brazil’s disease monitoring system, henceforth referred to as SINAN *(Sistema de Informação de Agravos de Notificação)* [23]. The second is aggregate Google search data from the Google Trends API. Informed consent for secondary analysis of this data for research purposes was not obtained as part of the data generation process for either the chikungunya case data or Google search data. However, all data is anonymous, analysed at low geographic granularity (city-level for the case data and state-level for the search data) and either provided as an aggregate weekly measure (in the case of the search data) or aggregated to weekly level for analysis (in the case of the chikungunya case count data). Approval for secondary analysis of this anonymous data was obtained from the University of Warwick’s Humanities and Social Sciences Research Ethics Committee (HSSREC application reference 42/19–20).

### Materials

We obtain anonymised chikungunya case data for the city of Rio de Janeiro through the InfoDengue project [9]. Our case data begins on 3 January 2016, shortly after the start of the first chikungunya epidemic in Rio de Janeiro, and ends on 5 January 2020. The raw data is case-level: for each case, we have access to a notification date and an entry date. The notification date is the date on which a doctor first diagnoses a chikungunya case. The entry date is the date that a suspected chikungunya case is entered into the surveillance system. Case confirmation is usually based on symptoms alone, as only 10% of cases are confirmed by laboratories. If a laboratory finds that a chikungunya case is falsely diagnosed, it is retroactively removed from the system.

Table 1 shows that case entry usually occurs well after notification; only 50% of notified cases are entered into the system within 2 weeks of notification. There are also some very long delays in the data, such that 26% of notified cases are still not entered after 4 weeks, and 13% of notified cases are still not entered after 8 weeks. We verify whether the delays are similar when only considering epidemic periods, as defined by the Moving Epidemic Method (MEM) [24]. The MEM analyses the frequency of cases across the sample to set a weekly case threshold, above which the week would be defined as an epidemic period. For our data, the epidemic threshold identified by the MEM is 104 cases. The second row of Table 1 shows that delays during epidemic periods are similar to delays when considering all of the data. However, Table 1 also reveals that 58% of weeks in this dataset fall in an epidemic. For this reason, we further examine the pattern of delays in different years, to help us understand whether delays are impacted by the size of the epidemic. We find little difference between the delays witnessed in individual years and those in the sample as a whole, regardless of the size or presence of an epidemic in each of the years. This suggests that any differences in the performance of the model in these different time periods are unlikely to be due to differences in the structure of the delays.

**Table 1:** Delays between chikungunya case diagnosis and entry into the disease surveillance system. There is often a long delay of weeks or months between initial diagnosis of a chikungunya case and entry of that case into the monitoring system. Varied delays make nowcasting on a weekly basis more difficult, as we lack complete data on both the most recent week and the weeks shortly preceding it. The first row describes the distribution of delays across the sample as a whole. Only 26% of cases are reported after 1 week, and 74% after 4 weeks. We further find evidence of a tail of cases with very long delays, with 13% of cases still not entered 8 weeks after notification. The second row describes the distribution of delays during epidemic periods, which are weeks in which the case count exceeds 104 cases. This is very similar to the sample as a whole. Similarly, we find that the distribution of delays in non-epidemic periods (third row) and the distributions of delays by year (fourth to seventh rows) do not differ greatly to the distribution of delays for the sample as a whole. This suggests that any differences in the performance of the model in these different time periods are unlikely to be due to differences in the structure of the delays.

		Mean percentage of cases entered after			
	Number of weeks	1 week	2 weeks	4 weeks	8 weeks
All periods	208	26%	50%	74%	87%
Epidemics	121	26%	51%	76%	90%
Non-epidemics	87	26%	48%	72%	84%
2016	52	31%	51%	68%	82%
2017	52	27%	49%	76%	88%
2018	52	24%	49%	78%	91%
2019	52	23%	49%	76%	89%

**Fig 1:**
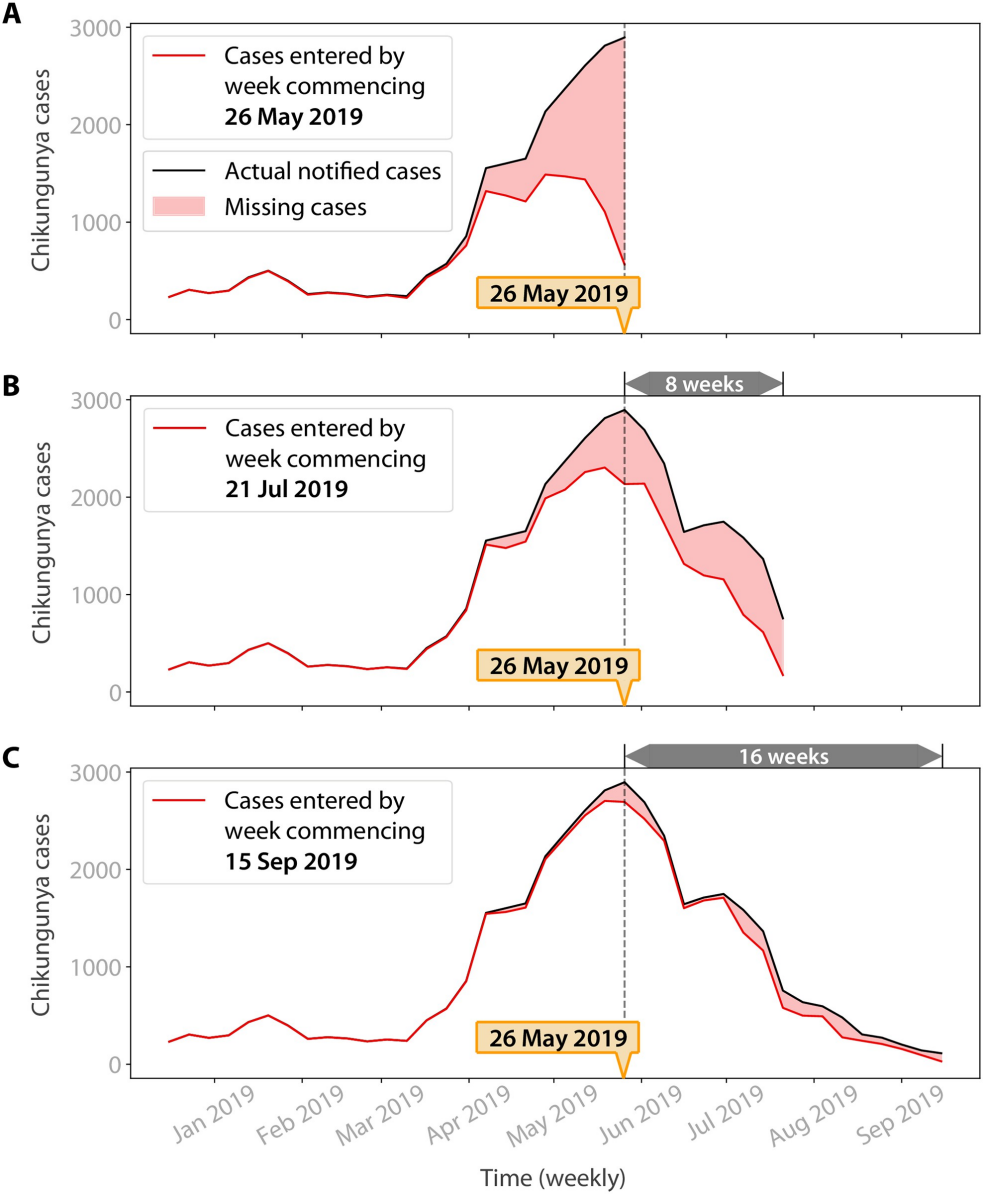
Chikungunya case data availability for an example week. Data on previous chikungunya cases arrives gradually and inconsistently. We illustrate this problem using the week commencing 26 May 2019: the peak of the 2019 epidemic in Rio de Janeiro. The black series show the true number of cases in each of the previous weeks. The red series show how many of these cases had been entered into the surveillance system by the end of the week. A) Only 20% of the 2,895 cases for the week commencing 26 May 2019 had been entered by the end of the week. B) The data is still very incomplete 8 weeks later, with only 74% of cases from the week commencing 26 May 2019 having been entered. C) The data is still not complete even after 16 weeks, with only 93% of cases from the week commencing 26 May 2019 having been entered. Data completeness is generally worse for more recent weeks, but this is not a consistent relationship. For example, by 15 September 2019, 79% of the cases in the week commencing 18 August 2019 had been entered, but only 58% of cases had been entered for the week commencing 11 August 2019, despite this being a week earlier.

To illustrate the length and inconsistency in reporting delays, Fig 1 shows a snapshot of data availability for the week commencing 26 May 2019. There were 2,895 diagnosed cases during the week, but these cases were entered into the system only gradually over the following weeks, with around 25% of cases still not entered after two months. At the end of the example week, the data on previous weeks was similarly incomplete, with completeness generally being worse for more recent weeks.

The fact that the case count data remains incomplete for several weeks poses a challenge for building an appropriate nowcasting model. Specifically, if we wish to estimate the total cases *X*_*t*_ at the end of week *t*, the lagged variable *X*_*t*−1_ will not be very informative, as this count will be artificially low due to incompleteness. It would be possible to mitigate this incompleteness by working with monthly data instead of weekly data. For example, if we wished to estimate the total cases *X*_*m*_ at the end of month *m*, the lagged variable *X*_*m*−1_ would reflect cases from over a month ago. Table 1 shows that the level of incompleteness would be much less for a variable lagged by a month, *X*_*m*−1_, than it would be for a variable lagged by a week, *X*_*t*−1_. However, delivering monthly estimates would leave policymakers working with infrequent updates on a disease situation which typically develops at much greater tempo.

To enable us to deliver weekly nowcasting estimates despite the case count data remaining incomplete for such lengthy periods, our time series modelling must therefore go beyond an approach that expects lagged data to be complete, in contrast to nowcasting approaches that have worked well in other areas of disease surveillance and beyond [14, 19, 25]. We return to this point in the *Methods* section.

Our second dataset reflects Google search behaviour, and is available at weekly resolution with no delay from the Google Trends API. We obtain weekly data on Google searches between January 2016 and January 2020. To retrieve data on searches related to chikungunya, we use Wikidata [26] to identify the Freebase topic ID for chikungunya (/m/01__7l), in line with an approach taken in previous work on dengue [17]. Limitations on the spatial granularity of data available from the Google Trends API mean that we need to retrieve data for the whole state of Rio de Janeiro, rather than just for the city. A key question for these analyses is therefore whether state-level search data will prove informative for city-level chikungunya case counts.

**Fig 2:**
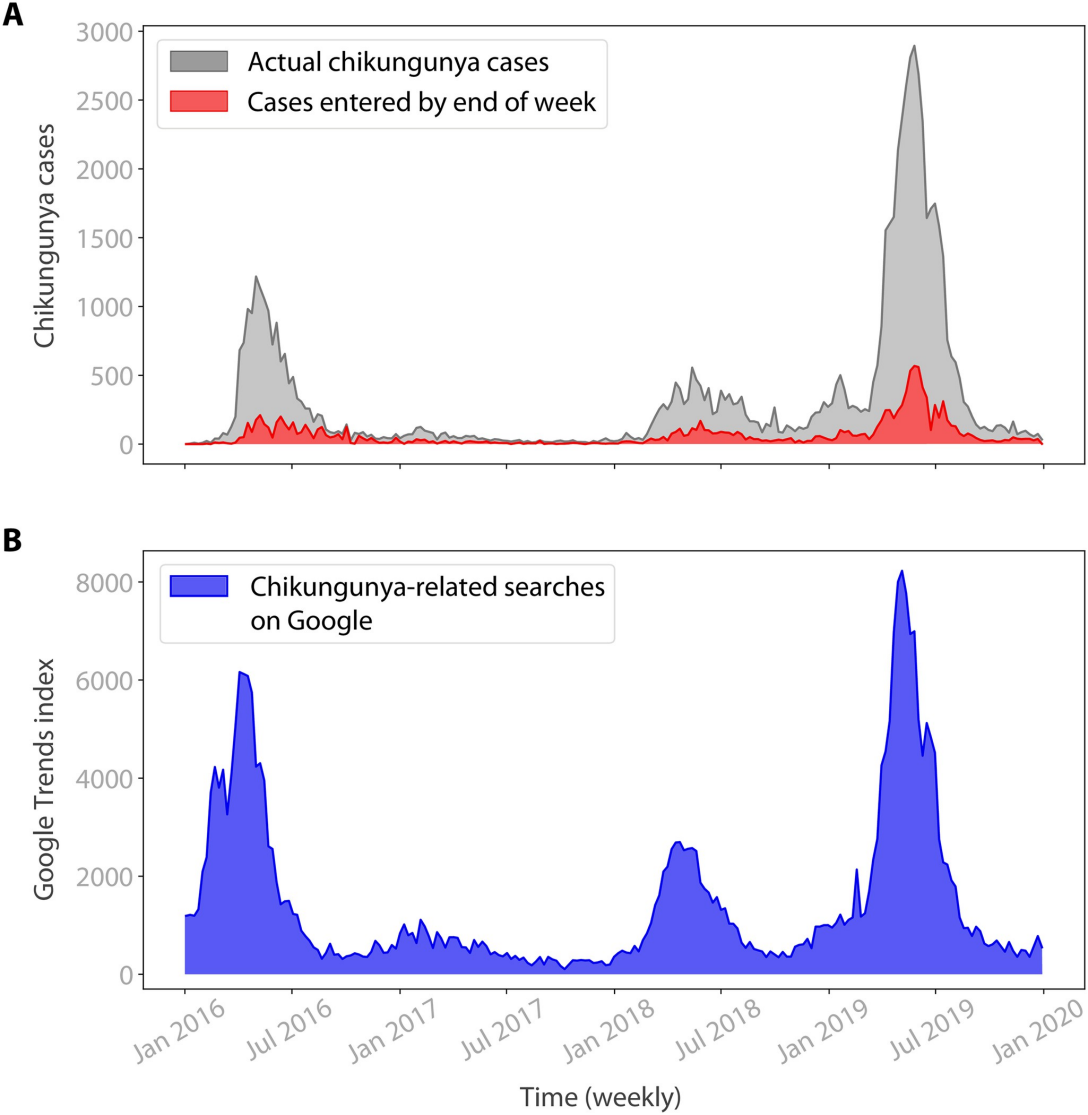
Comparing chikungunya case counts and Google searches over time. A) Time series of chikungunya cases per week in Rio de Janeiro, from 2016 to 2020. The grey series shows the number of cases diagnosed in each week (the notified cases). The red series shows the number of cases diagnosed in a given week that have been entered into the disease surveillance system by the end of that week. Entered cases are an inconsistent fraction of notified cases, and this issue is most severe during the large epidemics in 2016 and 2019. This makes estimating current chikungunya case counts from the official data alone particularly challenging. B) Time series of weekly Google searches for chikungunya-related terms, which are available in real-time. Visually, there is a strong correlation with the case count time series, with Google searches peaking during the large epidemics in 2016 and 2019. The size of the peaks in Google searches also seems to match the size of the epidemics, with the biggest peaks during the 2016 and 2019 epidemics. Google search data may therefore provide a rapid indicator of chikungunya case counts.

Fig 2 provides early evidence that, despite this limitation, spikes in Google searches for chikungunya may provide a rapid indicator of higher case counts. There is visually a strong correlation, with the peaks in Google searches occurring on or before the epidemic peaks in 2016, 2018 and 2019. The magnitude of spikes in Google searches are also visually a good fit for the magnitude of epidemics, with the biggest spikes in searches occurring during the 2016 and 2019 epidemics.

Weekly Google search data is reliably available in real-time: at the end of any given week, we have a complete record of search behaviour for that week. This provides a stark contrast to the official chikungunya case data that is available at the end of any given week, where we lack complete data on both the week that has just passed and previous weeks. Therefore, we hypothesise that Google search data may help us nowcast chikungunya case counts.

### Methods

Our objective is to estimate the current weekly case count *X*_*t*_, using only data available by the end of week *t*. This is the challenge faced by health professionals in a surveillance setting.

We compare the performance of three models. We first consider the performance of a statistical nowcasting model designed to estimate chikungunya incidence based on historic chikungunya data alone, whilst accounting for the incompleteness of recent data (the *baseline nowcasting model*). This baseline model is currently implemented in the InfoDengue system. We then investigate whether this baseline nowcasting model can be improved by additionally considering Google search data (the *nowcasting model using Google searches*). Finally, to provide real-world context for the performance of these statistical models, we consider a model that aims to capture the heuristic approach used by policymakers to mitigate against incompleteness of recent data, whereby data from the most recent weeks is simply disregarded (the *heuristic model*).

#### Baseline nowcasting model

To generate baseline nowcasts of chikungunya in Rio de Janeiro, we employ a nowcasting model developed for case count data that arrives gradually and inconsistently [21]. For each week, this model aims to estimate the number of cases that will be entered into the system with a given number of weeks delay, using data available in week *t*. Table 2 provides a stylised example of the data availability problem the model aims to address.

**Table 2:** Stylised example of chikungunya case count data availability for a given week. This matrix provides a stylised example of the chikungunya case count data available when nowcasting cases for a given week. In this example, we hold data from week 1 onwards and are currently in week 7. For ease of illustration, we assume here that the maximum delay in entering a case into the surveillance system is five weeks. Each row represents a previous week (*t*) of entered cases, and the column represents the entry delay (*d*) in weeks. For example, we can see that there were initially 15 cases entered into the system in week 2, 8 further cases after a delay of 1 week, 10 cases after a delay of 2 weeks, and so on. Case data is incomplete, not only for week 7 but also weeks 3 through 6. The incompleteness is usually worse the closer the week to the current period, so there is a running “triangle” of unknown case counts and associated delays to be estimated for previous weeks. Estimating each cell in the last row of this triangle yields a nowcast of the total case count for week 7. The method introduced by Bastos et al. [21] provides an approach for generating these estimates.

		Delay in weeks (*d*)						
		0	1	2	3	4	5	Total
Week (*t*)	1	10	12	6	4	1	1	34
	2	15	8	10	2	4	1	40
	3	19	9	13	5	2	?	?
	4	19	9	13	5	?	?	?
	5	17	25	11	?	?	?	?
	6	26	20	?	?	?	?	?
	7	39	?	?	?	?	?	?

To estimate total cases *X*_*t*_ in week *t*, we must therefore estimate how many cases will be entered with a delay of *d* weeks (*x*_*t*,*d*_), where

$$X_{t}=\sum_{d=0}^{D}x_{t,d}$$

For efficiency, in fitting the model we discard all cases for which case entry was delayed for over 26 weeks. We then set the maximum delay *D* to the number of weeks delay required to include 95% of the remaining cases in training, or 8 weeks if this is greater.

Following Bastos et al. [21], we assume *x*_*t*,*d*_ has a negative binomial distribution:

$$x_{t,d}∼NB\left(\lambda_{t,d},\phi \right)$$

We estimate the mean of this distribution, λ_*t*,*d*_, with the following specification:

$$\text{log}\;\left(\lambda_{t,d}\right)=\alpha +\beta_{t}+\gamma_{d}$$

where

- *α* is a time-invariant constant.
- *β*_*t*_ is a first order random walk (rw1) random effect $\beta_{t}∼N\left(\beta_{t-1},\sigma_{\beta }^{2}\right)$ capturing serial correlation in case counts. If we observe larger case counts in the previous week, we estimate a higher case count for the current week.
- *γ*_*d*_ is an rw1 random effect $\gamma_{d}∼N\left(\gamma_{d-1},\sigma_{\gamma }^{2}\right)$ capturing serial correlation in the number of cases reported with a given number of weeks delay. If we observe a greater number of cases with *d* − 1 weeks delay, we estimate a higher number of cases with *d* weeks delay too.

We fit the parameters for this specification using the Integrated Nested Laplace Approximation (INLA) method [27]. We estimate each *x*_*t*,*d*_ via sampling, which yields a posterior distribution of estimates for $X_{t}=\sum_{d=0}^{D}x_{t,d}$. This distribution provides a natural measure of uncertainty, with wider distributions implying greater uncertainty.

Using this model, we estimate the chikungunya case count in each week *t* >= 21 (in other words, from 22 May 2016 onwards) utilising an adaptive nowcasting procedure [14] as follows. We initially train a model with the first 20 weeks of data. The model then outputs a posterior distribution of estimated case counts for week 21. We record the difference between the mean case count estimate and the true case count as the model’s out-of-sample nowcast error. In each following week *t* > 21, we re-train the model with all available data at week *t*. Therefore, the model “adapts” over time [14]. Previous nowcasting studies where models were not retrained using the most recent available data led to overestimates of flu incidence [28].

In this analysis, we always train on all data prior to week *t* rather than using a fixed training window, following previous work on nowcasting dengue [17]. We discuss the pros and cons of this approach in the *Discussion*.

#### Nowcasting model using Google searches

We now define our nowcasting model using Google searches. This is similar to the baseline nowcasting model, but also includes Google search data *G*_*t*_ as a covariate:

$$\text{log}\;\left(\lambda_{t,d}\right)=\alpha +\beta_{t}+\gamma_{d}+\delta \;\text{log}\left(G_{t}\right)$$

where *δ* is a regression coefficient.

Google search data is fully available by the end of the week. We can therefore include *G*_*t*_ directly, rather than having to estimate it in the same way as the chikungunya case data.

#### Heuristic model

Finally, we operationalise the heuristic approach used by policymakers to mitigate against incompleteness of recent data. In the heuristic model, we disregard the last few weeks of incomplete data and instead use the number of notified cases from three weeks ago, *X*_*t*−3_, as an estimate of the current number of cases, *X*_*t*_.

The heuristic model provides important context for the performance of the statistical nowcasting models. Its inclusion is inspired by what Yang et al. term the “naive” model in their dengue nowcasting analyses [25] (see also [17]). We expect the baseline nowcasting model to perform much better than the commonly applied heuristic approach. We are therefore analysing whether Google search data can help us further improve the performance of a carefully chosen baseline nowcasting model that is well-suited to this nowcasting problem.

### Model comparison

Following previous work on nowcasting dengue [17], we calculate a range of metrics for our models to allow us to investigate both the error and uncertainty of our estimates. For all models, we report model error in terms of mean absolute error (MAE), where a lower MAE reflects more accurate estimates. We report uncertainty in terms of the mean 95% prediction interval width (MPI), where a smaller interval represents reduced uncertainty about model estimates. It is important to verify that any reduction in the size of the 95% prediction interval is not simply due to the interval becoming too narrow and no longer reliable. We therefore also report interval reliability, in terms of the percentage of true weekly case counts that fall within the 95% prediction interval for that week. 95% of true weekly cases falling within the 95% prediction interval represents good interval reliability. Finally, we report results both for the full period and when considering epidemic periods alone, as epidemics are likely to be particularly important for policymakers. We define epidemic periods using the 104 weekly case threshold previously calculated using the Moving Epidemic Method (MEM) [24].

## Results

**Fig 3:**
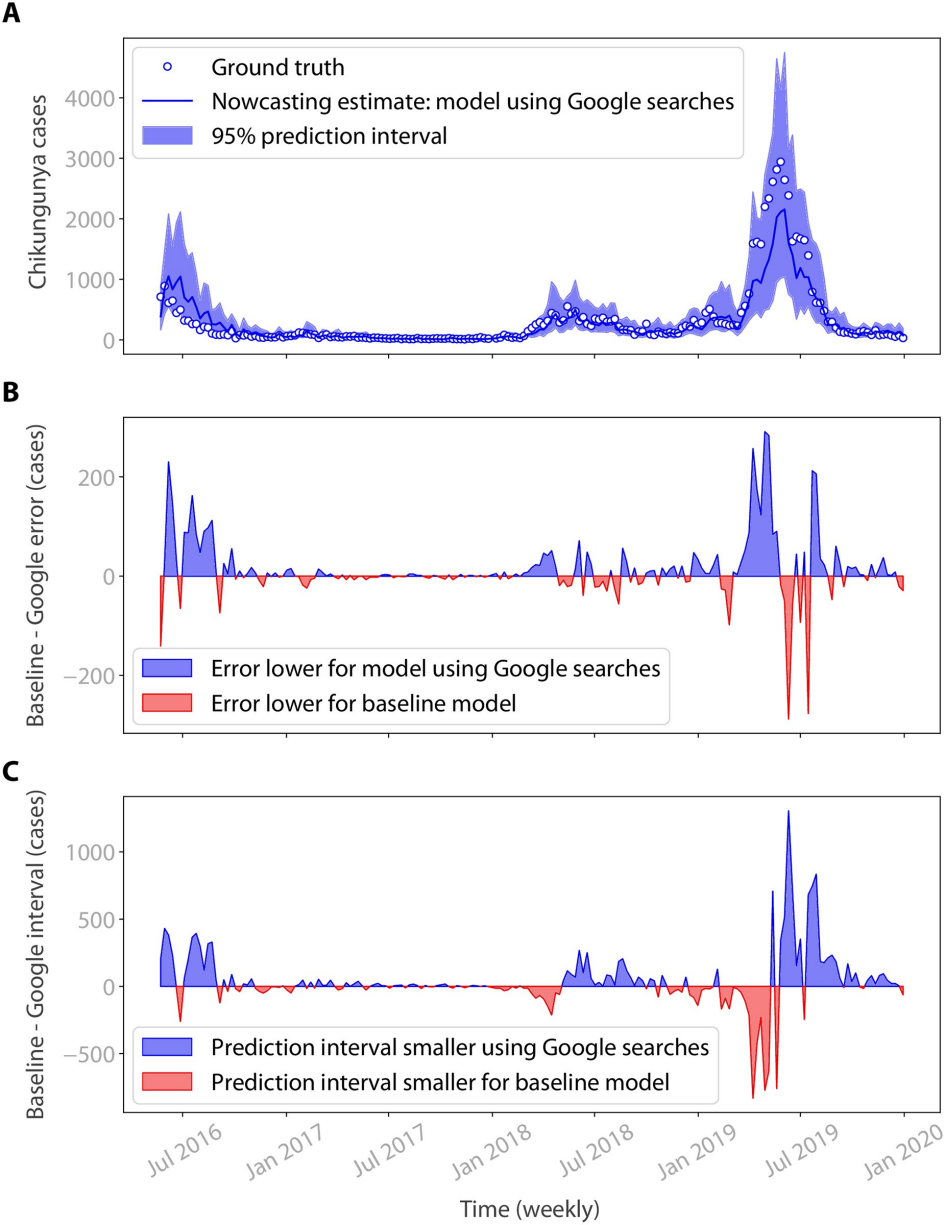
Performance of the nowcasting model using Google searches. A) Nowcast results over time for the model using data on Google searches for chikungunya. The model’s estimates are relatively accurate across the sample. Moreover, the ground truth rarely falls outside the 95% prediction intervals. B) A comparison of relative nowcast errors, in case numbers, between the baseline nowcasting model and nowcasting model using Google searches. Blue indicates that error is lower for the model using Google searches, and red indicates that error is lower for the baseline model. Overall the errors produced by the model using Google searches are lower, although there is some volatility around epidemic periods. C) A comparison of relative prediction interval width, in case numbers, between the baseline model and model using Google searches. The prediction interval produced by the model using Google searches is generally narrower, except for the period at the start of the 2019 epidemic. However, Table 4 shows that the baseline model prediction intervals are less reliable during an epidemic. For example, several of the true weekly case counts in the first half of the 2019 epidemic fall outside the baseline prediction interval (Fig A in S1 Appendix), but within the prediction interval produced by the model using Google searches.

Table 3 compares model error across the heuristic model, baseline nowcasting model and nowcasting model using Google searches. The baseline nowcasting model is much more accurate than the heuristic model, reducing MAE by 34% across the sample as a whole. Adding Google search data to the model further improves upon the baseline, reducing MAE by 41% relative to the heuristic model. Fig 3A shows that estimates produced by the model using Google searches are rarely far from the true case count.

**Table 3:** Comparison of error in chikungunya case count estimates for the heuristic model, baseline nowcasting model and nowcasting model using Google searches. We compare the mean absolute errors (MAEs) for the chikungunya nowcasts produced by the heuristic model, baseline model and model using Google searches. The first two columns show results across the sample as a whole, reported in number of cases and relative improvement in comparison to the heuristic model. The baseline nowcasting model is far more accurate than the heuristic model, reducing MAE by 34%. The model using Google searches improves further upon the baseline, reducing MAE by 41% relative to the heuristic model. The third and fourth column show results when only considering epidemic periods. Here we see that the error reductions offered by both the baseline model and model using Google searches increase further: the baseline model reduces MAE relative to the heuristic model by 35%, and the model using Google searches reduces MAE by 43%.

Model	All periods		Epidemics	
	MAE	Relative MAE	MAE	Relative MAE
Baseline	124.8	0.66	212.8	0.65
Google	110.9	0.59	187.7	0.57
Heuristic	187.9	1.00	327.0	1.00

In Table 3, we report the error of the model estimates during epidemics as well as across the full period. The improvement offered by the baseline nowcasting model over the heuristic model increases further during epidemics, reducing MAE by 35%. In turn, there is an increase in the improvement offered by the model using Google searches over the baseline model too, with the model using Google searches reducing MAE by 43% relative to the heuristic model. Fig 3B shows there are few periods during epidemics where the baseline model outperforms the model using Google searches. This is further corroborated when splitting the errors by year, as shown in Table A in S1 Appendix. The model using Google searches outperforms the baseline model during the years in which the chikungunya case count exceeds the epidemic threshold for many weeks (2016, 2018 and 2019). In 2017, the baseline model outperforms the model using Google searches, but both models display very low error rates.

Table 4 compares the uncertainty of the baseline model and model using Google searches in terms of the mean 95% prediction interval width (MPI). The heuristic approach does not allow an interval to be calculated. Fig 3A shows that ground truth weekly case counts very rarely fall outside the 95% prediction interval produced by the model using Google searches. Furthermore, there are no instances where the ground truth falls far outside the prediction interval.

**Table 4:** Comparison of uncertainty in chikungunya case count estimates for the baseline nowcasting model and nowcasting model using Google searches. We compare the mean prediction interval widths (MPIs) for nowcasts from the baseline model and model using Google searches. The heuristic model is omitted as this approach does not allow a prediction interval to be calculated. The first three columns show results across the sample as a whole. The first column is the MPI reported in number of cases; the second column is the MPI relative to the baseline nowcasting model; and the third column is the percentage of actual weekly case counts within the prediction interval. The nowcasting model using Google searches is more precise than the baseline nowcasting model, reducing MPI by 7%. It is also slightly more reliable, capturing 93% of actual weekly case counts relative to 91% for the baseline model. In Table C in S1 Appendix, we show that the slight overconfidence of the prediction intervals is largely driven by the first epidemic, where the model had little data to train on. The final three columns show similar results when considering epidemic periods only. While the intervals are much wider for both models, the model using Google searches reduces MPI by 8%. The intervals produced by the model using Google searches are also even more reliable relative to the baseline during epidemics, capturing 93% of weekly case counts relative to 88%.

Model	All periods			Epidemics		
	MPI	Relative MPI	% Correct	MPI	Relative MPI	% Correct
Baseline	524.2	1.00	91.0	873.6	1.00	88.1
Google	485.6	0.93	92.6	805.5	0.92	93.1

The nowcasting model using Google searches exhibits less uncertainty than the baseline nowcasting model, reducing MPI by 7%. While the prediction interval width increases for both models when considering epidemic periods alone, the intervals produced by the model using Google searches remain 8% smaller than the baseline intervals. This lower uncertainty holds for each year in the sample, with the model using Google searches reducing the mean prediction interval width by between 4% and 14% each year, as shown in Table B in S1 Appendix.

Finally, the nowcasting model using Google searches also produces more reliable prediction intervals than the baseline nowcasting model. Across the sample as a whole, the prediction interval produced by the model using Google searches captures 93% of the ground truth while the baseline model prediction interval only captures 91% (Table 4). This difference is larger during epidemics: the prediction interval produced by the model using Google searches captures 93% of the ground truth whereas the baseline model interval captures only 88%. The slight overconfidence of the intervals seems largely driven by the first epidemic, where the model had little data to train on (see Table C in S1 Appendix for results excluding the first epidemic).

Some of the periods where the baseline prediction interval is narrower than the prediction interval produced by the model using Google searches occur during epidemics, particularly in the first half of 2019 (Fig 3C). However, several of the true weekly case counts during the early 2019 epidemic fall outside the baseline prediction interval (Fig A in S1 Appendix), but within the interval produced by the model using Google searches (Fig 3A). Therefore, the narrower baseline prediction intervals may not be as reliable during an epidemic, which further favours the Google nowcasting model.

Visual examination of Fig 3B suggests that the nowcasting model using Google searches may be particularly effective relative to the baseline nowcasting model prior to the epidemic peak. To investigate this further, we analyse results from 2018 and 2019, as we do not have data from the onset of the 2016 epidemic, and weekly cases only briefly exceeded the epidemic threshold in 2017. For both 2018 and 2019, we consider weeks in which the case count is above the epidemic threshold of 104 cases. We split this data into the period prior to the epidemic peak and the period after the epidemic peak. We find that the errors produced by the model using Google searches are 16% lower than the baseline in the period prior to the 2018 epidemic peak (Table D in S1 Appendix), and 15% lower prior to the 2019 peak (Table E in S1 Appendix).

Similarly, visual comparison of the baseline model’s performance in Fig A in S1 Appendix to the performance of the model using Google searches in Fig 3A suggests that the prediction intervals produced by the model using Google searches may be more reliable in the period prior to the epidemic peak. The prediction intervals produced by the model using Google searches are larger in this period: 15% larger in 2018 and 14% larger in 2019 (Tables F and G in S1 Appendix). However, the actual case counts fall within the baseline prediction intervals only 82% of the time in the period prior to the 2018 epidemic peak and 86% of the time prior to the 2019 peak. By contrast, the actual case counts fall within the intervals produced by the model using Google searches 100% of the time in the periods prior to both the 2018 and 2019 epidemic peaks (Tables F and G in S1 Appendix).

Overall then, the nowcasting model using Google searches appears to deliver better performance than the baseline nowcasting model in the periods before epidemic peaks, displaying lower errors and greater reliability of the prediction intervals. There is less of a difference in model performance during the period following the epidemic peak. Following the 2018 epidemic peak, the errors for the model using Google searches are 7% lower than the baseline model errors, but following the 2019 peak, the errors for the model using Google searches are 4% higher (Tables D and E in S1 Appendix). However, the prediction intervals produced by the model using Google searches are 11% narrower than the baseline following the epidemic peak in 2018, and 21% narrower in 2019. There is no difference in the frequency with which the actual case counts fall within the prediction intervals: for both models, this is 100% following both the 2018 and 2019 peaks (Tables F and G in S1 Appendix).

The strong performance of the model using Google searches during epidemic onset periods may be particularly helpful for providing early warning of epidemics to policymakers. Again, we define epidemic periods using the 104 weekly case threshold previously calculated using the Moving Epidemic Method (MEM) [24] as applied in the InfoDengue project [9]. We investigate which model provides the most timely detection of this threshold being crossed at the beginning of an epidemic. We consider detection of the 2018 epidemic as a case study, as the case count does not sink below 104 cases for more than two continuous weeks before the 2019 epidemic. In 2018, the actual case count crosses the threshold of 104 cases in epidemiological week 9. In contrast, the heuristic model does not detect threshold crossing until week 15 of the epidemiological year. The baseline model detects the epidemic earlier, estimating that the threshold is crossed in week 12. However, the model using Google searches produces the closest estimate, detecting threshold crossing in week 11. The model using Google searches therefore provides four weeks of early warning relative to the heuristic approach, which monitors only entered cases. These four weeks could have been crucial for policymakers seeking to intervene early in order to limit the spread of chikungunya.

## Discussion

We have analysed whether Google search data can help improve chikungunya nowcasting in Rio de Janeiro, Brazil. Chikungunya in Rio de Janeiro is seasonal, with attack rates varying from year to year. Early warnings of bad outbreaks are important for delivering timely interventions.

Data on chikungunya cases is usually entered into the surveillance database with a significant delay after diagnosis, making decisions less timely. These delays are also inconsistent, increasing the challenge of estimating current chikungunya case counts from official data alone. Here, we have examined the performance of three approaches to delivering weekly estimates of chikungunya incidence in Rio de Janeiro whilst mitigating against delays and incomplete data. These are a heuristic approach, frequently applied by policymakers in practice, where data on the last few weeks is simply disregarded; a baseline nowcasting model, as currently implemented in the InfoDengue system, where a statistical approach is employed to model the varying delays in the data; and a nowcasting model using Google searches, which augments the baseline nowcasting model with rapidly available Google search data. We evaluate the error of the models’ estimates of chikungunya incidence when using the data that was available at the end of each week. For the baseline nowcasting model and nowcasting model using Google searches, we also examine the size of the prediction intervals accompanying the estimates, to understand how certain policymakers could be of the estimates delivered.

We find that both the baseline model and the model using Google searches outperform the heuristic approach by some margin. Importantly, while the baseline model performs well, we find that including Google search data reduces both nowcast error and uncertainty relative to the baseline. Our analyses show that including Google search data reduces nowcast errors between May 2016 and December 2019. When considering only epidemic periods, which are particularly important for policymakers, we find a similar reduction. We further find that including Google search data reduces nowcast uncertainty, reducing prediction intervals by 8% during epidemics and 7% across the sample as a whole. Finally, including Google search data may make prediction intervals more reliable during epidemics. We find that, during epidemics, the prediction interval produced by the model using Google searches captures 93% of weekly case counts compared to 88% for the baseline interval. Our model can be used in practice to generate weekly estimates, despite the significant and varied delays in the entry of chikungunya case count data.

In this analysis, we always train the baseline nowcasting model and nowcasting model using Google searches on all data from earlier weeks, rather than using a fixed training window. Updating the model to include the most recent data is important, as the predictive relationship between search data and disease incidence may change over time [14, 28]. However, previous work on dengue found little evidence that discarding past data in training leads to a reduction in error [17]. As epidemics are infrequent, using all previous data also helps avoid a situation where the epidemics are lost from the training data due to a shorter training window. However, one important advantage of using a shorter fixed size training window is a reduction in computation time [29]. This advantage accumulates if estimates are being produced for thousands of cities in parallel, as is currently the case for the InfoDengue platform. Future work could further examine the performance of the chikungunya nowcasting approaches outlined here with a fixed size training window, to ensure that this parameter delivers the optimal combination of reduced error and rapid computation.

The analysis we present here focuses on the city of Rio de Janeiro. However, the Google search data is for the state of Rio de Janeiro, rather than the city. Further research could build on our results by testing whether they hold for other cities in the state, whose Google search behaviour may be less well correlated with the state’s overall search behaviour. Promising initial indications are provided by previous analyses for dengue that demonstrate that state-level Google data can still help reduce error and uncertainty, even in smaller cities [30].

Our methods could also be extended to other areas of Brazil, or other arbovirus-prone regions, such as India, which have experienced chikungunya outbreaks [8]. Inhabitants of other regions may have a different relationship with the internet. For example, they may use it less frequently to gather information on illness. It would be valuable to analyse whether Google search data is still effective for nowcasting in such scenarios. Future work should therefore consider a wider range of chikungunya-prone regions and states beyond the state of Rio de Janeiro.

A key limitation of the analysis we present here is the relatively short length of the case count time series available for training. This spans four years, and hence approximately 200 weekly data points. Both the baseline model and model using Google searches overestimate the 2016 epidemic. We suggest that this is due to the fact that this epidemic falls at the beginning of the sample, when little model training has been completed. Both models also underestimate the 2019 epidemic. This is likely to be due to that model training through two smaller outbreaks in 2017 and 2018. Nevertheless, the true data points for the 2019 epidemic still fall within the 95% prediction interval of the model using Google data. As further data arrives, it will be possible to continue to monitor the performance of the proposed nowcasting model.

In the analysis described here, we have also only considered one real-time data source. Future nowcasting research could include other data sources, whether measuring other online activity [17, 31], or properties of the external environment related to arbovirosis incidence, such as the weather [32].

Finally, we note that other arboviruses spread by the *Aedes aegypti* mosquito in Brazil, such as dengue and Zika, exhibit similar symptoms. This can cause challenges for medical practitioners in diagnosis. Similarly, increases in Google searches for one of these diseases could be driven by an increase in cases of another [17]. Future research could jointly model their incidence, which may be more effective than modelling them independently. If so, policymakers would be able to respond more quickly to epidemics across a range of diseases.

## Supporting information

S1 Appendix Supplementary analyses of model performance. (PDF)
