# Supplementary material for: Faster indicators of chikungunya incidence using Google searches
Source: PLoS Negl Trop Dis. 2022 Jun 9;16(6):e0010441. doi: 10.1371/journal.pntd.0010441 (PMC9182328; doi:10.1371/journal.pntd.0010441)
Supplement: S1 Appendix — (PDF) [file pntd.0010441.s001.pdf]

S1 Appendix:

Faster indicators of chikungunya incidence using Google searches

Sam Miller[1,2*], Tobias Preis[1,2], Giovanni Mizzi[1], Leonardo Soares Bastos[3], Marcelo Ferreira da Costa Gomes[3], Flávio Codeço Coelho[4,5], Claudia Torres Codeço[3], Helen Susannah Moat[1,2*]

1 Data Science Lab, Behavioural Science, Warwick Business School, University of Warwick, Coventry, United Kingdom
2 The Alan Turing Institute, London, United Kingdom
3 Programa de Computação Científica, Fundação Oswaldo Cruz, Rio de Janeiro, Brazil
4 Escola de Matemática Aplicada, Fundação Getulio Vargas, Rio de Janeiro, Brazil
5 Institute of Global Health, University of Geneva, Geneva, Switzerland

* smiller@turing.ac.uk (SM); Suzy.Moat@wbs.ac.uk (HSM)

Table A. Comparison of model errors by year. We compare the mean absolute errors (MAEs) for the nowcasts produced by the heuristic model, baseline model and model using Google searches. In each year in which the chikungunya case count exceeds the epidemic threshold for many weeks (2016, 2018 and 2019), the model using Google searches outperforms the baseline model. In 2017, the baseline model outperforms the model using Google searches, but both models display very low error rates. The poor performance in 2016 of the baseline model and model using Google searches relative to the heuristic approach is likely to be a result of the models being initialised during the 2016 epidemic, and therefore lacking training data.

Period		Baseline	Google	Heuristic
2016	MAE	199.8	167.8	103.6
	Relative MAE	1.93	1.62	1.0
2017	MAE	9.2	10.0	18.1
	Relative MAE	0.50	0.56	1.0
2018	MAE	54.2	48.0	112.2
	Relative MAE	0.48	0.43	1.0
2019	MAE	264.8	239.8	485.4
	Relative MAE	0.55	0.49	1.0

Table B. Comparison of model intervals by year. We compare the mean prediction interval widths (MPIs) for nowcasts from the baseline model and model using Google searches. The heuristic model is omitted as this approach does not allow a prediction interval to be calculated. The model using Google searches displays less uncertainty than the baseline model in each year, with smaller MPIs. This advantage ranges from a 4% to 14% reduction in the size of the MPIs, as can be seen by examining the relative MPIs. Note that the intervals for both models capture a low proportion of true weekly case counts in 2016, likely due to the lack of training data at this point.

Period		Baseline	Google
2016	MPI	650.8	558.3
	Relative MPI	1.0	0.86
	% correct	65.6	62.5
2017	MPI	75.8	71.9
	Relative MPI	1.0	0.95
	% correct	98.1	96.2
2018	MPI	337.4	323.4
	Relative MPI	1.0	0.96
	% correct	96.2	100
2019	MPI	1081.5	1016.8
	Relative MPI	1.0	0.94
	% correct	94.2	100

Table C. Prediction intervals excluding the first epidemic. In the Results section and Table 4 in the main text, we note that the 95% prediction intervals for both models do not contain the true case counts as often as they should. We suspect this is due to the first epidemic, where the model must make predictions given little training data. This table shows prediction intervals excluding those produced for weeks during the first epidemic, as we analyse intervals for dates after 1 September 2016 only. The intervals produced by both the baseline model and the model using Google searches are more reliable once the first epidemic is excluded, with the ground truth falling within the baseline model interval 94% of the time, and the interval produced by the model using Google searches 96% of the time.

	All periods			Epidemics		
Model	MPI	Relative MPI	% correct	MPI	Relative MPI	% correct
Baseline	468.2	1.00	94.2	817.7	1.00	93.1
Google	444.1	0.95	96.0	773.7	0.95	98.9

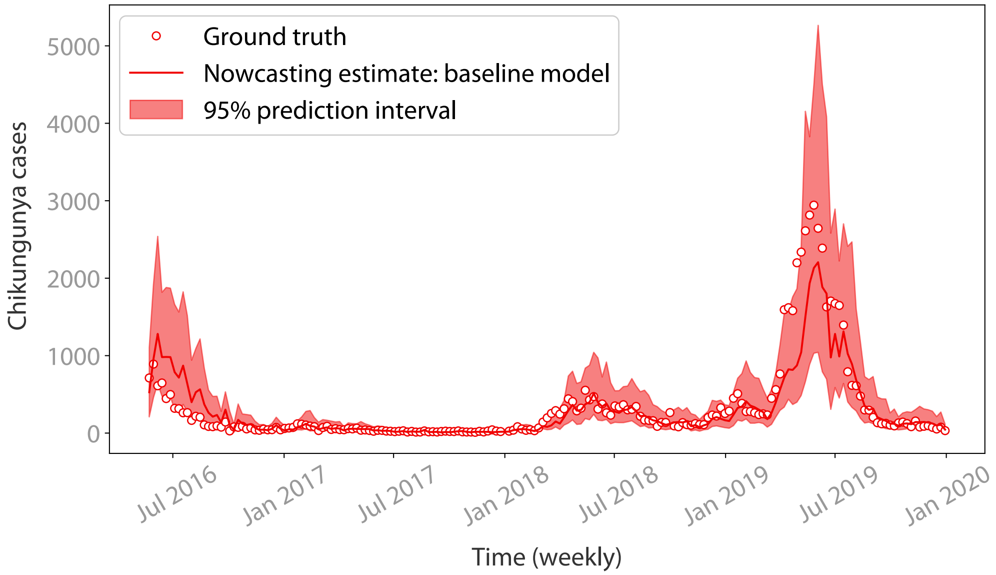

Fig A. Chikungunya case count estimates and prediction intervals produced by the baseline model. In Fig 3 in the main text, we observe that there are some periods where the baseline model prediction interval is narrower than the interval produced by the model using Google searches. However, we also note that these may be periods where the baseline interval is not large enough to cover the area in which the ground truth data falls 95% of the time. This figure shows baseline model performance over time. At the start of the 2019 epidemic, there are several weeks where the ground truth falls outside the baseline model interval. These fall in the timespan where the baseline model intervals are narrower than the intervals produced by the model using Google searches. Therefore, when the baseline intervals are narrower, they may not be a reliable indicator of the true chikungunya case count, which further favours the model using Google searches.

Table D. Errors during the 2018 epidemic. The errors produced by the model using Google searches are 16% lower than the baseline model errors prior to the epidemic peak, but only 7% lower after the epidemic peak.

	Before peak		Post peak	
Model	MAE	Relative MAE	MAE	Relative MAE
Baseline	99.6	0.44	50.0	0.51
Google	83.5	0.37	46.6	0.48
Heuristic	226.9	1.00	97.1	1.00

Table E. Errors during the 2019 epidemic. The errors produced by the model using Google searches are 15% lower than the baseline model errors prior to the epidemic peak, but 4% higher after the epidemic peak.

	Before peak		Post peak	
Model	MAE	Relative MAE	MAE	Relative MAE
Baseline	447.6	0.57	182.7	0.47
Google	380.8	0.49	189.6	0.49
Heuristic	783.7	1.00	385.4	1.00

Table F. Prediction intervals during the 2018 epidemic. Before the epidemic peak, the intervals produced by the model using Google searches are 15% larger than those produced by the baseline model. However, the baseline model intervals only contain the true weekly case count 82% of the time. By contrast, the intervals produced by the model using Google searches correctly contain the true case count 100% of the time. After the epidemic peak, the intervals produced by the model using Google searches are 11% smaller than those produced by the baseline model, with no difference in the frequency with which the intervals contain the true weekly case count.

	Before peak			Post peak		
Model	MPI	Relative MPI	% correct	MPI	Relative MPI	% correct
Baseline	383.3	1.00	81.8	406.3	1.00	100.0
Google	439.4	1.15	100.0	360.3	0.89	100.0

Table G. Prediction intervals during the 2019 epidemic. Before the epidemic peak, the intervals produced by the model using Google searches are 14% larger than those produced by the baseline model. However, the baseline model intervals only contain the true weekly case count 86% of the time. By contrast, the intervals produced by the model using Google searches are correct 100% of the time. After the peak, the intervals produced by the model using Google searches are 21% smaller than those produced by the baseline model, with no difference in the frequency with which the intervals contain the true weekly case count.

	Before peak			Post peak		
Model	MPI	Relative MPI	% correct	MPI	Relative MPI	% correct
Baseline	1150.3	1.00	85.7	1369.0	1.00	100.0
Google	1309.2	1.14	100.0	1081.6	0.79	100.0
